# Impaired Function of Peripherally Induced Regulatory T Cells in Hosts at High Risk of Graft Rejection

**DOI:** 10.1038/srep39924

**Published:** 2016-12-23

**Authors:** Takenori Inomata, Jing Hua, Antonio Di Zazzo, Reza Dana

**Affiliations:** 1 Schepens Eye Research Institute, Massachusetts Eye and Ear Infirmary, Department of Ophthalmology, Harvard Medical School, Boston, MA, USA

## Abstract

Regulatory T cells (Tregs) are crucial for allograft survival. Tregs can be divided into thymus-derived natural Tregs (tTregs) and peripherally-derived induced Tregs (pTregs). Here, we determine whether the suppressive function of Treg subsets is hampered in hosts who are at high risk for rejecting their graft. To induce graft beds that promote high risk of transplant rejection, intrastromal corneal sutures were placed two weeks prior to the transplant procedure in mice. We demonstrate that in high-risk recipients the frequencies and function of pTregs (but not tTregs) are suppressed. Reduced function of pTregs correlated with decreased expression of CTLA-4, interleukin-10, and transforming growth factor-β. Adoptive transfer of pTregs from mice at low risk of subsequent graft rejection is able to rescue graft survival in recipients that are at high risk of rejecting their grafts. Our data suggest that impaired function of pTregs, but not tTregs, mediates the loss of immune tolerance and promotes allograft rejection.

Regulatory T cells (Tregs) maintain immune homeostasis by dampening inflammatory responses toward self- and alloantigens^1^,^2^,^3^, and therefore play a crucial role in allograft survival. Studies in solid organ transplantation have shown that Treg-based therapies can be effective in promoting long-term tolerance to skin and heart grafts not only in experimental animals^4^,^5^, but also in human subjects^6^,^7^. Studies have suggested that Foxp3^+^ natural Tregs (nTregs; thymic-derived Tregs [tTregs]) and peripherally-induced Tregs (pTregs) can act in concert to promote tolerance^8^,^9^,^10^, but alloantigen-specific pTregs are thought to be the principal mediators of allograft tolerance^11^,^12^,^13^. While these and other studies have shed light on the antigen-specificity of Tregs on graft survival, much of the results are limited to Tregs that are artificially induced *in vitro* through controlled alloantigen exposure, or using transgenic strains with altered immune systems^8^,^9^,^10^,^14^,^15^. This has left important questions open, especially regarding the differential contributions of Treg subsets to the natural allotolerance that is developed *in vivo*. Understanding the natural involvement and precise function of Treg subsets in allotolerance is essential for effective development of Treg-based strategies in transplantation. Tregs are a heterogeneous population; recently, animal studies have shown that natural thymus-derived tTregs and some (auto) antigen-specific Tregs are neuropillin-1^+^ (Nrp-1^+^) while in transplantation alloantigen-specific peripherally-induced pTregs^16^ are Nrp-1^−^17,18. This differentiation has permitted studies evaluating these distinct Treg subsets in a variety of immunopathologies.

In all forms of transplantation, certain recipients are known to be at particularly high risk of rejecting their grafts. In the case of corneal transplantation, which is the most commonly performed tissue grafting procedure^19^, success rates are very high in uninflamed (so-called ‘low-risk’) host beds (as seen in simple corneal scars)^20^. Similarly, in the murine model of corneal transplantation close to one-half of allografts survive indefinitely without any local or systemic immunomodulatory treatment, reflective of the immune privileged status of corneal grafts. In contrast, host factors such as graft bed inflammation and neovascularization lead to high rejection rates^21^ regardless of the magnitude of immune suppression^20^,^22^. Thus, understanding the underlying mechanisms that enable, or abrogate, normal Treg function is essential for optimizing immunomodulatory strategies in transplantation, especially in hosts at high risk of rejection. In the current study, we used a model of corneal transplantation^23^,^24^ to delineate the differential function and susceptibility of tTregs and pTregs from allograft recipients – low-risk hosts with normal immune homeostatic mechanisms who develop allotolerance naturally, and high-risk hosts with inflamed graft beds who are prone to swift rejection of their transplants.

## Results

### Treg dysfunction in high-risk transplantation

**Figure 1:**
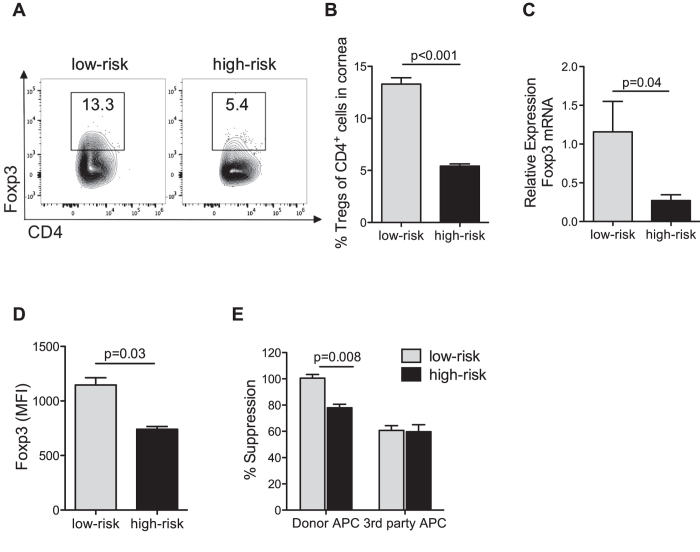
Regulatory T cell dysfunction in high-risk graft recipients. Corneal grafts were transplanted onto inflamed high-risk or control (‘low-risk’) host beds, and their corneas and draining lymph nodes (dLNs) were analyzed 14 days post-transplantation. (**A**, **B**) Corneal grafts were harvested and digested with collagenase D. Single cell suspensions were stained for Foxp3 and CD4, and analyzed using flow cytometry. 5 corneas per group were pooled for each analysis; data shown are representative of 3 independent experiments. (**C)** Corneal grafts were harvested, mRNA was isolated, and Foxp3 expression was analyzed using real-time PCR 14 days post-transplantation. n = 5. (**D**) DLNs were harvested, each DLN was stained for Foxp3 and CD4, and analyzed separately using flow cytometry. Mean fluorescence intensity (MFI) of Foxp3 levels by Tregs of low-risk and high-risk graft recipients was assesssed. N = 5 mice/group, data shown are representative of 3 independent experiments. (**E**) Treg suppression assay showing Treg suppressive function of Tregs isolated from DLNs of high-risk vs. low-risk recipients 14 days post-transplantation. Suppression of naïve BALB/c CD4^+^CD25^−^ conventional T cell proliferation by Tregs was assessed following exposure to C57BL/6 (donor) or C3H (third party) APCs. All data were obtained from n = 5 mice/group and representative data from three independent experiments are shown. *p* values are calculated using the Mann-Whitney test and error bars represent SEM.

Transplantation was performed onto recipients with quiescent low-risk and inflamed high-risk graft beds. Grafted corneas were harvested to determine Treg migration to the graft site 14 days after corneal transplantation. Treg frequencies (Fig. 1A,B) and Foxp3 expression (Fig. 1C) were reduced in corneas from high-risk recipients compared to recipients at low-risk for rejection. Ipsilateral draining lymph nodes (dLNs) are the principal sites of allosensitization^25^; thus we compared Foxp3 expression and Treg functionality in the dLNs of high-risk vs. control low-risk graft recipients. Foxp3 protein expression in Tregs from dLNs of high-risk graft recipients was reduced compared to low-risk graft recipients (Fig. 1D). Tregs isolated from dLNs of high-risk recipients and cultured with Tconv cells displayed ~20% less suppressive function compared to Tregs isolated from low-risk control recipients (Fig. 1E). When donor-specific (C57BL/6) APCs were used to stimulate T cell proliferation, only Tregs from low-risk control recipients showed suppressive function; with third party C3H APCs, both high-risk and low-risk host-derived Tregs displayed a comparably low suppressive function at ~60% (Fig. 1E).

### Dysfunctional pTregs express reduced immunoregulatory molecules and increased inflammatory cytokine

**Figure 2:**
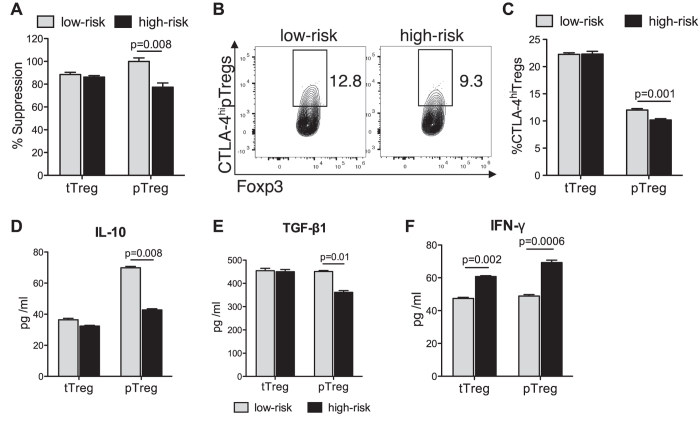
Function of pTregs and their differential expression of cytokines and co-immunosuppressive molecules. Corneal transplantation was performed in low and high-risk recipients, and draining lymph nodes (dLNs) were isolated 14 days post-surgery. For further analyses separate single cell suspensions for each DLN were prepared, n = 5/group. (**A**) pTregs and tTregs were FACS sorted from the dLNs of graft recipients according to their Nrp1 expression; pTregs = Nrp1^−^, tTregs = Nrp-1^+^. The suppressive potential of pTregs and tTregs on CD4^+^CD25^−^ conventional T cell proliferation in the presence of C57BL/6 APCs was compared using a Treg suppression assay. (**B**) Dot plot showing frequencies of CTLA-4^hi^ pTregs of dLNs from high-risk and low-risk recipients. (**C**) Flow cytometry analysis showing frequencies of CLTA-4^hi^ tTregs and pTregs in the dLNs of high-risk and low-risk recipients. (**D–F**) pTreg and tTregs were isolated from the draining lymph nodes of high-risk and low-risk recipients, cultured and the expression levels of (**D**) IL-10, (**E**) TGF-β1, and (**F**) IFN-γ were analyzed in the supernatant by ELISA. All data were obtained from n = 5 mice/group and represent data from three independent experiments. *p* values are calculated using the Mann-Whitney test and error bars represent SEM.

Alloantigen-specific peripherally-induced pTregs have been defined as Nrp-1^−^26,27. Here, we FACS sorted Nrp1^+^ tTregs and Nrp-1^−^ pTregs to assess their suppressive function on Tconv cell proliferation *in vitro*. We found that pTregs from high-risk recipients showed ~20% less suppressive function than pTregs from low-risk recipients (Fig. 2A). To determine how pTreg dysfunction in hosts prone to rejection may fail to induce allotolerance, we evaluated the expression of several key Treg regulatory molecules and cytokines critical for their function. First, we assessed the expression of the co-inhibitory molecule CTLA-4 by pTregs and tTregs, and noted that pTregs from high-risk recipients have lower frequencies of CTLA-4^hi^ pTregs whereas tTregs from high- and low-risk recipients show no difference (Fig. 2B and C). pTreg frequencies and their Foxp3 expression in the DLNs of low-risk and high-risk graft recipients were analyzed before and 14 days after transplantation. Corneal suturing did not affect pTreg frequencies or Foxp3 expression, but transplantation into these high-risk graft beds reduced their Foxp3 expression compared to transplants into low-risk (not sutured) graft beds (Supplemental Fig. 1A–C). Comparing pTreg frequencies and Foxp3 expression high-risk acceptors vs. rejctors 14 days post-transplantation showed no difference in pTreg frequencies but reduced Foxp3 expression in rejectors (Supplemental Fig. 1D and E). Then, we measured levels of IL-10, TGF-β1, and IFN-γ in the supernatant of sorted and cultured pTregs and tTregs using ELISA. IL-10 (Fig. 2D) and TGF-β1 (Fig. 2E) expression levels were significantly reduced in pTregs from high-risk recipients. In contrast, IFN-γ expression by both pTregs and tTregs from high-risk recipients was increased (Fig. 2F). In grafted corneas, we found less IL-10 but increased IFN-γ and IL-12 expression in high-risk recipients (Supplemental Fig. 2).

### Adoptive transfer of functional pTregs restores allotolerance

**Figure 3:**
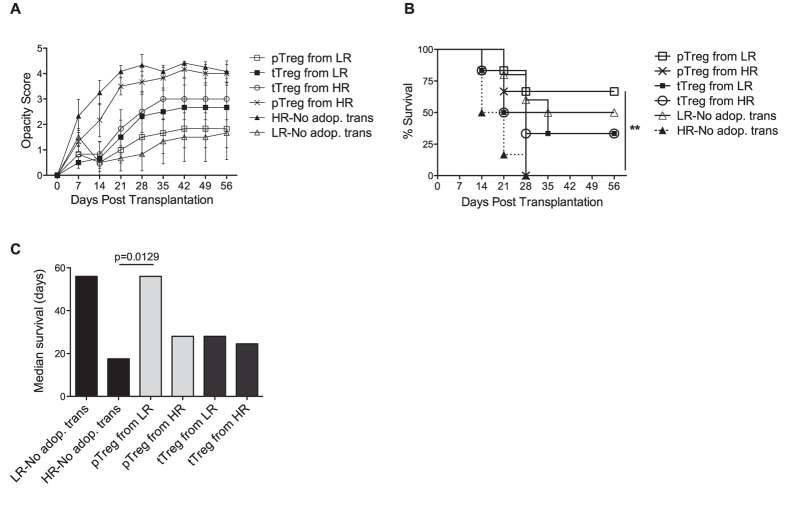
Effect of pTreg and tTreg adoptive transfer on allograft survival in high-risk recipients. pTregs and tTregs were isolated from the draining lymph nodes (dLNs) of high-risk and low-risk (LR) recipients at day 14 post-transplantation, and 1 × 10^5^ cells were intravenously injected into high-risk (HR) recipients 18 hours post-transplantation (n = 6/group). High-risk and low-risk recipients without transfer served as controls (No adop. trans). (**A**) Opacity scores and (**B**) graft survival were monitored for up to 8 weeks post-transplantation (**p < 0.01). HR hosts transferred with LR pTregs had a significantly higher survival (p = 0.006) and reduced opacity scores (**p < 0.01) than HR recipients without adoptive transfer. Log-rank test. (**C**) The median graft survival shows that only pTregs from LR recipients significantly improved graft survival in HR recipients to levels seen in LR control recipients. Mann-Whitney Test, n = 6–10 mice/group.

To investigate whether normal allotolerance can be restored in high-risk hosts, we adoptively transferred pTregs or tTregs isolated from low-risk or high-risk recipients to high-risk recipients soon (18 hours) after transplantation. Our data showed that pTregs isolated from high-risk recipients were incapable of preventing allograft rejection, whereas pTregs from low-risk recipients prolonged graft survival and reduced graft opacity even in hosts that were considered at high risk for rejecting their allografts (Fig. 3A,B). Of note, the adoptive transfer of pTregs from low-risk grafted controls to high-risk recipients enhanced median graft survival to levels seen normally with low-risk hosts without transfer (Fig. 3C). Although tTregs from both high-risk and control recipients prolonged graft survival, they were less efficient than the highly functional pTregs derived from low-risk recipients in promoting graft survival.

## Discussion

Tregs have attracted broad interest for their role in supporting allograft survival. Corneal allografts normally enjoy very high survival rates due to tolerogenic mechanisms that account for ocular immune privilege^28^; however, in settings of graft site inflammation (‘high-risk’ transplantation) the risk of a prompt rejection becomes a near absolute certainty^20^,^29^,^30^.

In accord with previous studies reporting reduced Treg frequencies in solid organ graft rejection^31^,^32^,^33^, we show herein decreased Treg frequencies at the graft site of high-risk grafted hosts. We further observed decreased Foxp3 expression in high-risk corneas and by Tregs from DLNs of high-risk recipients. Because Foxp3 is the key transcription factor for Tregs and its expression level correlates with Treg suppressive function^23^,^34^, Tregs from high-risk recipients with less Foxp3 expression display decreased inhibitory functions *ex vivo*.

It is recognized that Foxp3^+^ Tregs are comprised of multiple subsets, and it has been suggested that pTregs control antigen-specific immune responses in the periphery, whereas tTregs maintain general immune homeostasis^8^,^9^,^10^. Here, we show that pTregs from high-risk recipients show reduced suppressive function compared to pTregs from low-risk recipients. In accord with our previous study^23^, we found that pTregs from high-risk recipients with rejected corneas express less Foxp3 compared with high-risk acceptors (Supplemental Fig. 1D, E). In this context, and given our data, we propose that pTregs induce peripheral tolerance for allografts under physiologically normal conditions, whereas graft site inflammation leads to pTreg instability or dysfunction after transplantation, which in turn promotes graft rejection. Although sutures in the cornea may induce local inflammation, we see similar pTreg frequencies and Foxp3 expression in the DLNs of mice before transplantation with sutured and non-manipulated corneas (Supplemental Fig. 1).

Tregs exert their suppressive function via expression of inhibitory cell surface molecules (e.g., CTLA-4)^35^ and through production of immunoregulatory cytokines, such as IL-10 and TGF-β^14^,^36^. In high-risk hosts we observe reduced CTLA-4, IL-10, and TGF-β expression by pTregs, which correlates with their reduced suppressive function both *in vivo* and *in vitro.* In contrast, the acquisition of IFN-γ by Tregs isolated from high-risk hosts suggests not only impaired regulatory function but also an adopted proinflammatory function, which may further imperil allograft survival. In accord, we found decreased IL-10 and increased IFN-γ and IL-12 mRNA expression in grafted corneas of high-risk recipients (Supplemental Fig. 2), indicating that Treg dysfunction in high-risk recipients with inflamed beds is caused at the local site.

Our adoptive transfer experiments demonstrate that transferred functional pTregs from control low-risk recipients displayed the highest capacity to rescue graft survival, similar to levels seen in control low-risk recipients. In previous studies, we have reported on the relevance of the graft bed microenvironment on APC maturation and migration, and subsequent allosensitization^21^,^37^. Based on the results presented, we propose that the graft microenvironment cannot only enhance allosensitization through increased APC maturation and migration, as we have reported previously^21^,^37^, but can also dictate the functional specificities of pTregs, which in turn control allograft fate. Given the known functional plasticity of Tregs^38^, these data also suggest the potential feasibility of local graft-site cytokine manipulation to alter microenvironmental cues that regulate pTreg function and ultimately promote graft survival.

## Materials and Methods

### Animals

Six-week-old BALB/c (H-2d), C57BL/6 (H-2b), and C3H/He male mice were purchased from Charles River Laboratories (Wilmington, MA, USA). All animal experiments were approved by the Institutional Animal Care and Use Committee of the Schepens Eye Research Institute, and were conducted in accordance with the Association for Research in Vision and Ophthalmology (ARVO) statement for the Use of Animals in Ophthalmic and Vision Research.

### Suture-induced Inflamed Graft Bed Preparation

Inflamed, neovascularized (‘high-risk’) host beds were created as detailed previously^24^. Briefly, three intrastromal sutures were placed into the central cornea using 11–0 nylon sutures (AB-0550S, MANI, Tochigi, Japan) 14 days before corneal transplantation to induce host bed inflammation and angiogenesis, thus rendering the host at high risk of rejecting their allograft^21^. Mice with unmanipulated (clear, non-vascularized) corneal host beds at low-risk of graft rejection served as controls.

### Allogeneic Corneal Transplantation

For allogeneic corneal transplantation C57BL/6 corneas were grafted onto BALB/c host beds as detailed elsewhere^24^. Briefly, the central cornea (2-mm diameter) was excised from a donor C57BL/6 mouse using scissors (Vannas; Storz Instruments, San Dimas, CA). The graft bed was prepared by excising a 1.5-mm site in the central cornea of a BALB/c mouse. The donor button was then placed onto the recipient bed and secured with eight interrupted 11–0 nylon sutures. Corneal sutures were removed 7 days after surgery. Graft survival was evaluated for 8 weeks using a slit-lamp biomicroscope. We used a standard opacity-grading (range, 0–5+) scheme to define rejection^39^; corneas with an opacity score of 2+ for two consecutive examinations were considered rejected. In order to evaluate Treg function before graft rejection set in, clear grafts from both recipient groups were analyzed at day 14.

### Isolation of Corneal Cells

Single-cell suspensions were prepared from the corneal samples by collagenase digestion, as previously described^40^. In brief, corneas were digested in RPMI media (Lonza, Walkersville, MD) containing 2 mg/ml collagenase type IV (Sigma-Aldrich, St. Louis, MO) and 2 mg/ml DNase I (Roche, Basel, Switzerland) for 60 min at 37 °C, and then filtered through a 70-μm cell strainer.

### Flow Cytometry

Ipsilateral draining lymph nodes (dLNs) and corneas (n = 5/group) were harvested and single-cell suspensions were prepared. To analyze corneal cells we pooled 5 corneas for each analysis; DLNs were analyzed separately. Each analysis was repeated twice. To avoid non-specific staining, cells were blocked with an anti-FcR blocking antibody (eBioscience, San Diego, CA, USA), and then stained with the following antibodies: anti-CD4 FITC (RM4–5), anti-CD25 PE (PC61), anti-Foxp3 PECy7 (FJK-16s) (BioLegend, San Diego, CA, USA), anti-Neuropilin-1 Alexa700 (FAB566N, R&D Systems, Minneapolis, MN, USA) and anti-CTLA-4 APC (UC10–4B9, BioLegend). Control samples were stained with appropriate isotype-matched control antibodies. Stained cells were examined using an LSRII Flow Cytometer (BD Biosciences, Franklin Lakes, NJ, USA), and the results were analyzed using FlowJo software X 10.0.7 (FlowJo LLC, Ashland, OR, USA).

### Cell Sorting

For *in vitro* analysis CD4^+^CD25^−^ conventional T cells (Tconv) and CD4^+^CD25^+^ Tregs from BALB/c mice and antigen-presenting cells (APCs) from C57BL/6 and C3H mice were isolated by magnetic-assisted cell sorting (MACS) using Treg and CD90.2 (depletion) isolation kits according to the manufacturers’ instructions (Miltenyi Biotec, Bergisch-Gladbach, Germany), respectively. For the *in vitro* studies and adoptive transfer experiments CD4^+^CD25^+^Nrp-1^+^ Treg (tTreg) and CD4^+^CD25^+^ Nrp-1^−^ Treg (pTreg) cells were sorted using a BD FACSAria™ III sorter (BD Biosciences, Franklin Lakes, NJ, USA).

### Treg Suppression Assay

Conventional T cells (Tconv; 1 × 10^5^) isolated via MACS sort from the dLNs of naïve BALB/c mice were cocultured with Tregs (5 × 10^4^) from transplant recipients (14 days post-transplantation), T cell-depleted allogeneic splenocytes from C57BL/6 or C3H mice (1 × 10^5^), and 1 μg/ml anti-CD3 antibody (145–2C11, BioLegend) for 3 days. Proliferation was measured using the BrdU incorporation assay (EMD Millipore, Billerica, MA, USA), and percent suppression was calculated using the following formula: % suppression = [(Tconv proliferation without Tregs – Tconv proliferation with Tregs)/ (Tconv proliferation without Tregs)] ×100. Percent suppression of Tregs from low-risk recipients cocultured with Tconv and donor APCs was set 100%.

### RNA Isolation, RT-PCR, and Real-Time PCR

RNA was isolated (RNeasy Micro Kit; Qiagen, Valencia, CA, USA) from 5 mice per group (dLNs or cornea) 14 days post-transplantation and reverse transcribed (Superscript Kit; Invitrogen, Carlsbad, CA, USA). Real-time PCR was performed using a PCR mix (Taqman Universal PCR Master mix; Invitrogen) and preformulated primers for Foxp3 (Mm00475156_ml), IL-10 (Mm00439614_m1), IFN-γ (Mm00801778_m1), IL-12 (Mm00434165_m1), and Glyceraldehyde-3-Phosphate Dehydrogenase (GAPDH; Mm999999_gl) (Applied Biosystems, Austin, TX, USA). Results were analyzed by the comparative threshold cycle method, using GAPDH as an internal control. Real-Time PCR was repeated three times for each cytokine with triplicates for each group.

### Enzyme-linked immunosorbent assay

Tregs from 5 mice/group were isolated 14 days post-transplantation via MACS sort from the dLNs of high-risk and low-risk graft-recipients, FACS sorted for pTregs and tTregs, and protein expression of IL-10, TGF-β1, and IFN-γ was analyzed in the supernatants after stimulation with PMA and inomomycin (IL-10 and IFN-γ) or LPS (TGF-β1) for 24 hours using ELISA kits according to the manufacturers’ instructions (eBioscience).

### Treg Adoptive Transfer

First, dLNs of high-risk and low-risk graft recipients were isolated at 14 days post-transplantation. After FACS sorting, 1 × 10^5^ pTregs or tTregs were suspended in 100 μl phosphate buffered saline (PBS) and transferred intravenously (i.v.) to high-risk host 18 hours post-transplantation. High-risk and low-risk recipients with no transfer served as controls. Allograft survival and opacity scores were monitored in each group (n = 6–10 mice/group) for up to 8 weeks post-transplantation.

### Statistical analysis

All animals were evaluated by a masked observer unaware of the source of adoptively transferred cells. Mann-Whitney test was used to compare means between groups. The One-Way Anova test was used to analyze opacity scores. Kaplan-Meier analysis was used to construct survival curves, and log-rank test was used to compare corneal graft survival. Data are presented as mean ± standard error of mean and considered statistically significant at *p* < 0.05.

## Additional Information

**How to cite this article**: Inomata, T. *et al*. Impaired Function of Peripherally Induced Regulatory T Cells in Hosts at High Risk of Graft Rejection. *Sci. Rep.* **6**, 39924; doi: 10.1038/srep39924 (2016).

**Publisher's note:** Springer Nature remains neutral with regard to jurisdictional claims in published maps and institutional affiliations.

## Supplementary Material

Supplemental Information
