# Supplementary material for: Impaired Function of Peripherally Induced Regulatory T Cells in Hosts at High Risk of Graft Rejection
Source: Sci Rep. 2016 Dec 23;6:39924. doi: 10.1038/srep39924 (PMC5180229; doi:10.1038/srep39924)

Impaired Function of Peripherally Induced Regulatory T Cells in Hosts at High Risk of Graft Rejection

Takenori Inomata,[1] Jing Hua,[1] Antonio Di Zazzo, and Reza Dana[*]

Supplementary Information

Supplemental Fig 1. pTreg frequencies and Foxp3 expression. (**A-C**) Corneal sutures were place into graft beds 2 weeks before transplantation to generate high-risk graft beds. Low-risk graft beds remained unmanipulated. Here, we analyzed draining lymph nodes of hosts before (ungrafted) and after (Day 14) transplantation, and corneas with (high-risk) and without (low-risk) sutures. (A and B) Flow cytometry analysis showing pTreg (Nrp-1- Foxp3+ CD4+) frequencies. (C) Mean Fluorescence Intensity (MFI) of Foxp3 in pTregs is shown for before and after transplantation in low-risk and high-risk graft recipients. (**D and E**) pTreg frequencies (D) and their Foxp3 expression (E) were analyzed 14 days post-transplantation in high-risk recipients with accepted and rejected corneas.

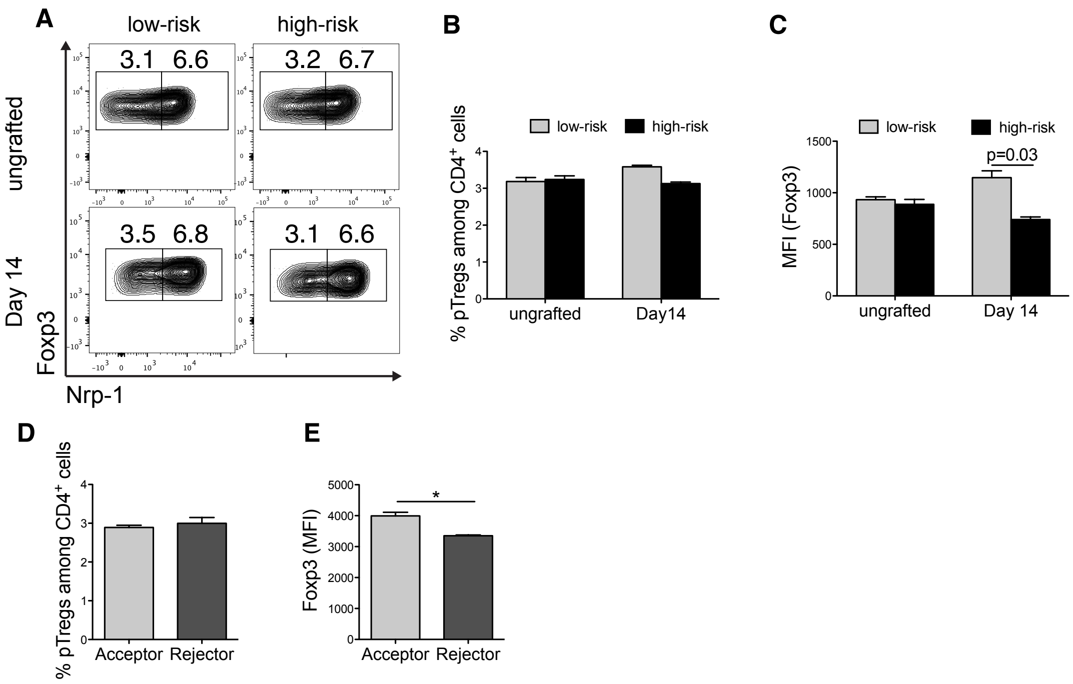

Supplemental Fig 2. Cytokine expression in the cornea of high-risk and control recipients.

Real-time PCR analysis of grafted corneas was performed 14 days post-transplantation. The mRNA cytokine expression of (**A**) IL-10, (**B**) IFN-γ, and (**C**) IL-12 was measured. Data from three independent experiments are shown. *p* values are calculated using the Mann-Whitney test and error bars represent SEM.

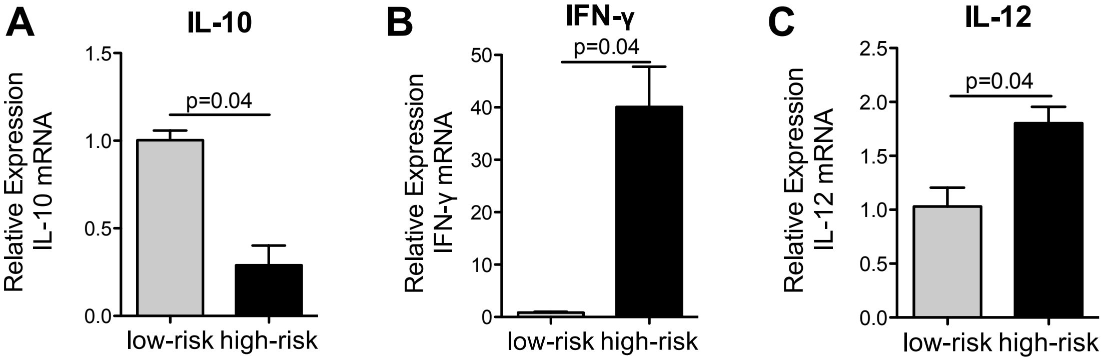

Supplement: Supplemental Information [file srep39924-s1.pdf]
